# Clinical Outcomes Associated with Screw Loosening in S2 Alar-Iliac Fixation in Adult Spinal Deformity

**DOI:** 10.3390/jcm14061881

**Published:** 2025-03-11

**Authors:** Yasuhiro Nagatani, Hiroaki Nakashima, Tokumi Kanemura, Mikito Tsushima, Hiroyuki Tomita, Kazuaki Morishita, Hiroki Oyama, Sadayuki Ito, Naoki Segi, Jun Ouchida, Ippei Yamauchi, Yukihito Ode, Yuya Okada, Shiro Imagama

**Affiliations:** Department of Orthopedic Surgery, Nagoya University Graduate School of Medicine, Nagoya 466-8560, Japan; yasuhiro.dragons9@gmail.com (Y.N.); spinesho@vmail.plala.or.jp (T.K.); hiro_tomi_1031@yahoo.co.jp (H.T.); pochi_pochi_pochity@yahoo.co.jp (K.M.); hiroki_o0831@yahoo.co.jp (H.O.); ito.sadayuki.w9@f.mail.nagoya-u.ac.jp (S.I.); naoki.s.n@gmail.com (N.S.); yaip0411@yahoo.co.jp (I.Y.); colon8102@gmail.com (Y.O.); okada.yuya.0219a@gmail.com (Y.O.); imagama.shiro.v4@f.mail.nagoya-u.ac.jp (S.I.)

**Keywords:** adult spinal deformity, sacral alar-iliac screw loosening, Japanese Orthopaedic Association back pain evaluation questionnaire

## Abstract

**Purpose**: To explore the clinical outcomes associated with screw loosening after S2 alar-iliac (SAI) screw fixation for adult spinal deformity (ASD). Overview of the literature: SAI fixation is widely used in ASD corrective surgery; despite the biomechanical advantages of this screw, complications such as screw loosening remain a significant concern. **Methods**: We retrospectively reviewed 27 patients undergoing corrective surgery for ASD using SAI as the distal fixation point at a single institution between February 2013 and February 2018. Patients were divided into non-loosening (N) and loosening (L) groups based on radiological evidence of screw loosening (radiolucent area of 1 mm around the screw) and compared for demographic characteristics, bone mineral density (T-score), spinal alignment parameters, and patient-reported outcome using the Japanese Orthopaedic Association Back Pain Evaluation Questionnaire (JOABPEQ) scores. **Results**: Screw loosening was observed in 78% of patients (n = 21/27); however, there were no statistically significant differences between the N and L groups in terms of the preoperative and 5-year postoperative JOABPEQ scores (*p* > 0.05 across all domains) and spinal sagittal alignment (*p* > 0.05). The improvements achieved through corrective surgery were largely maintained regardless of the presence of screw loosening. **Conclusions**: SAI screw loosening is a common sequela in ASD surgery; however, its loosening might not affect long-term clinical outcomes. Therefore, the choice of fixation should be individualized based on patient factors such as age and bone quality.

## 1. Introductions

Adult spinal deformity (ASD) represents a significant clinical challenge owing to its complex characteristics and the intricacies involved in its surgical management. Schwab et al. associated sagittal spinal parameters with patients’ disability outcomes and reported their thresholds [1]. Because the global sagittal alignment plays a crucial role in maintaining physical function [2], the importance of the global sagittal alignment has been emphasized. To maintain proper alignment after ASD corrective surgery, rigid distal fixation of the spine is important. Especially for the L5/S junction, a high rate of pseudoarthrosis or implant failure has been reported [3]. Previous studies have shown that long construct of the spine and fusion at the sacrum need pelvic fixation to prevent these surgical complications [4].

Pelvic fixation using S2 alar-iliac (SAI) screws is a beneficial option for distal fixation in long fusion surgery for ASD. Several studies have explored the role of SAI screws in achieving spinopelvic fixation and spinal biomechanics. SAI acts as a buttress to prevent withdrawal of the S1 screw and contributes to stabilization and bony fusion in the lumbosacral region. Nakashima et al. [5] and Iijima et al. [6] highlighted the biomechanical advantages of SAI screws, such as their alignment with the S1 screw and the reduced need for extensive hardware.

SAI screws offer several advantages over traditional iliac screws. Because of their low profile, SAI screws could reduce the rates of screw prominence and contribute to fewer hardware failures [7,8].

Despite these biomechanical benefits, the long-term stability of SAI screws, particularly in older patients with compromised bone quality, remains a concern.

Previous studies have documented the incidence of screws loosening in various fixation constructs [9,10].

The caudal screw loosening might cause poor outcomes. Banno et al. [11] reported that IS loosening caused lower fusion rates and insufficient correction after surgery, but little is known about SAI screws. S2AI screws penetrate the sacroiliac joint without bone fusion, so they might cause several complications related to the screws. Furthermore, these reports showed a follow-up period of about 2 years postoperatively; few papers have reported on long-term follow-up. So, the clinical implications of such loosening, particularly regarding long-term outcomes, remain unclear. We hypothesized that screw loosening would be associated with poor outcomes with long-term follow-up.

Therefore, the objective of this study was to identify factors associated with SAI screw loosening after pelvic fixation and to determine the impact of SAI screw loosening on clinical outcomes. First, we examined the frequency of SAI screw loosening following fixation surgery for ASD and performed comparative analyses to elucidate patient-related factors associated with screw loosening. Second, we investigated the relationship between SAI screw loosening and clinical factors.

## 2. Materials and Methods

Study design and setting: This retrospective analysis included patients undergoing corrective surgery for ASD at a single institution between February 2013 and February 2018.

**Figure 1:**
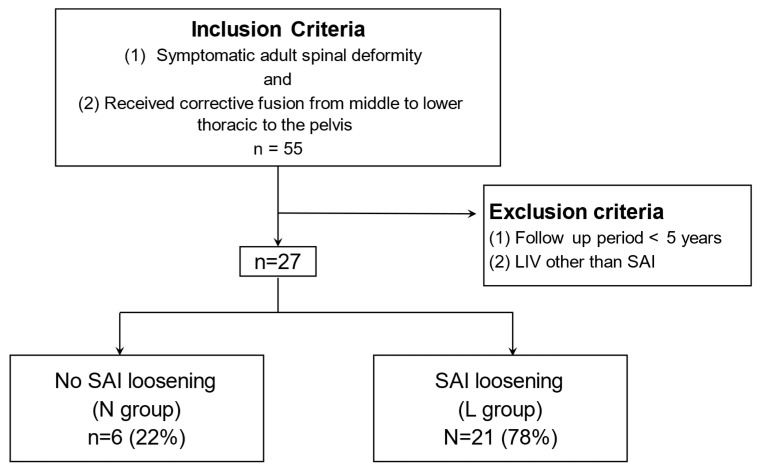
A total of 27 patients who underwent corrective surgery for an adult spinal deformity from 2013 to 2018 were included in the study.

**Figure 2:**
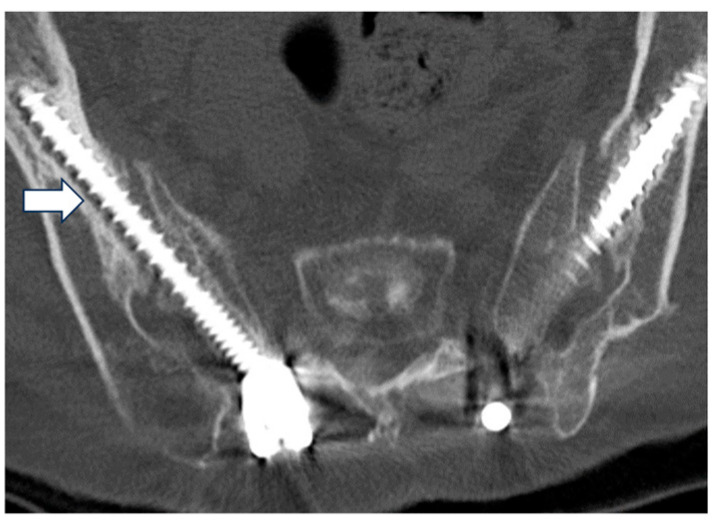
Assessment of the radiolucent area around the S2 alar-iliac (SAI) screw (white arrow).

Participants: We retrospectively reviewed 55 patients operated on during the aforementioned study period. Inclusion criteria were as follows: (1) patients with adult spinal deformity; (2) patients who met at least one of the following radiographic criteria (Cobb angle > 20°, sagittal vertical axis (SVA) > 5 cm, pelvic tilt (PT) > 25°, or thoracic kyphosis (TK) > 60°); (3) patients who received corrective fusion from the middle to lower thoracic to the pelvis. Exclusion criteria were as follows: (1) follow up period < five years; (2) patients without SAI screws as the primary anchoring mechanism. Accordingly, we included 27 patients in this study (Figure 1). The 27 patients were divided into 2 groups based on the presence or absence of SAI screw loosening that could influence long-term clinical outcomes and alignment. Screw loosening was defined based on the presence of a radiolucent area of 1 mm around the screw observed on computed tomography scans performed five years after surgery (Figure 2) [5]. Screw loosening was determined by two expert surgeons, and those with different results were determined by discussion. Radiological bony fusion at L5/S was also evaluated using CT reconstruction images. We judged whether bony fusion had been achieved with continuous bony bridging on sagittal and coronal CT scans. There were 6 patients (22%) who showed no signs of screw loosening and constituted the “N” group, while the remaining 21 patients (78%), demonstrating screw loosening, were attributed to the “L” group.

Variables: Between the N and L groups, we assessed multiple variables pre- and post-operation at five years including age; sex; body mass index (BMI); bone mineral density measured by femoral T-score; intraoperative factors (operative time and blood loss); spinal alignment parameters assessed using spine full-length standing X ray, such as SVA, TK, lumbar lordosis (LL), PT, sacral slope (SS), and pelvic incidence (PI); and the Japanese Orthopaedic Association Back Pain Evaluation Questionnaire (JOABPEQ) [12]. JOABPEQ is composed of five sections: pain-related disability, lumbar function, walking ability, social life function, and psychological well-being; the visual analog scale (VAS) was used for low back pain, pain in buttocks and lower limbs, and numbness in buttocks and lower limbs. Furthermore, patients were classified based on the L5/S fusion at five years, and the JOABPEQs were compared between patients with fusion (n = 22) and non-fusion (n = 5).

## 3. Statistical Analysis

We used the EZR software (version 1.6-6) for statistical analyses (Saitama Medical Center, Jichi Medical University, Shimotsuke-shi, Japan) [13]. The results were expressed as mean ± standard deviation or frequency and percentages (as applicable). Differences between the N and L groups were compared using Fisher’s exact test for categorical variables and the Mann–Whitney U test for continuous variables. A *p*-value of less than 0.05 was considered statistically significant. The results were expressed as mean ± standard deviation (SD) or as percentages where applicable.

## 4. Results

The mean age of patients was 69.2 ± 2.3 and 72.3 ± 7.5 years (*p* = 0.03), the mean BMI was 23.9 ± 3.4 and 23.0 ± 3.7 (*p* = 0.06), the mean T-score was 0.60 ± 1.8 and −1.08 ± 1.4 (*p* = 0.63), operative time was 631.2 ± 105.2 and 625.9 ± 136.7 min (*p* = 0.94), intraoperative blood loss was 1795.2 ± 976.3 and 1769.2 ± 842.2 mL (*p* = 0.95), L5/S bony fusion was observed in 5 patients (83.3%) and in 17 patients (81.0%) (*p* = 0.56), implant failure (rod fracture) occurred in 2 patients and 3 patients (N vs. L group) (Table 1). There was no reoperation in this study.

The two groups were comparable in terms of the preoperative JOABPEQ scores across various domains, namely pain-related disability (25.0 ± 13.9 vs. 41.7 ± 31.4; *p* = 0.32), lumbar function (33.3 ± 35.4 vs. 54.8 ± 22.5; *p* = 0.16), walking ability (30.3 ± 25.7 vs. 14.3 ± 27.2; *p* = 0.31), social life function (20.3 ± 22.4 vs. 25.4 ± 23.2; *p* = 0.70), psychological well-being (32.8 ± 13.9 vs. 36.6 ± 25.3; *p* = 0.78) and VAS scores (low back pain: 65.0 ± 41.9 vs. 68.8 ± 30.0; *p* = 0.82; pain in buttocks and lower limbs: 76.6 ± 23.0 vs. 69.3 ± 29.5; *p* = 0.61; numbness in buttocks and lower limbs: 76.6 ± 26.3 vs. 66.1 ± 34.2; *p* = 0.53) (N vs. L group).

Five years after surgery, still there were no differences between the two groups regarding JOABPEQ scores (N vs. L: pain-related disability, 39.3 ± 44.3 vs. 44.9 ± 34.0, *p* = 0.79; lumbar function, 35.5 ± 22.0 vs. 32.7 ± 24.9, *p* = 0.84; walking ability, 41.0 ± 14.9 vs. 31.9 ± 34.2, *p* = 0.65; social life function, 49.3 ± 11.8 vs. 38.7 ± 20.6, *p* = 0.35; psychological well-being, 46.3 ± 9.2 vs. 42.7 ± 18.3, *p* = 0.72). The mean visual analog scale (VAS) scores for low back pain, pain in buttocks and lower limbs, and numbness in buttocks and lower limbs before surgery were comparable between the N and L groups (*p*= 0.82, 0.61, and 0.53, respectively). The VAS scores for numbness in the buttocks and lower limbs were 56.8 ± 22.8 vs. 27.2 ± 21.0 (*p* = 0.01), for low back pain were 23.4 ± 16.5 vs. 28.4 ± 26.4 (*p* = 0.70), and for pain in the buttocks and lower limbs were 24.6 ± 24.8 vs. 32.6 ± 27.5 (*p* = 0.56) at 5 years post-operation (N vs. L group) (Table 2). SAI loosening might not be associated with clinical outcome at the 5-year follow-up.

Furthermore, comparison of the bony fusion at L5/S and the JOABPEQ showed no significant difference (pain-related disability, 38.0 ± 54.1 vs. 44.8 ± 32.8; lumbar function, 52.7 ± 29.0 vs. 29.5 ± 21.5; walking ability, 45.3 ± 48.6 vs. 31.6 ± 27.4; social life function, 50.7 ± 15.5 vs. 39.1 ± 19.7; psychological well-being, 56.0 ± 10.5 vs. 41.0 ± 16.6; low back pain, 29.1 ± 22.9 vs. 18.0 ± 34.7; pain in buttocks and lower limbs, 32.9 ± 25.8 vs. 21.0 ± 33.3; numbness in buttocks and lower limbs, 37.2 ± 23.4 vs. 11.8 ± 16.5, *p* > 0.05). With respect to spinal alignment, PT and PI showed statistical significance (PT, 19.1 ± 7.7 vs. 33.4 ± 8.8, *p* = 0.003; PI, 49.7 ± 8.7 vs. 64.1 ± 12.8, *p* = 0.01) (Table 3).

Regarding the preoperative spinal sagittal alignment, no statistically significant differences were observed between the two groups (N vs. L groups (*p*-value)). Mean SVA: 67.8 ± 43.3 mm vs. 98.4 ± 61.5 mm (*p* = 0.30); mean TK: 25.2° ± 17.6° vs. 23.6° ± 12.3° (*p* = 0.82); mean LL: 25.9° ± 17.7° vs. 22.0° ± 16.1° (*p* = 0.64); mean PT: 26.2° ± 6.5° vs. 32.3° ± 8.4° (*p* = 0.14); mean SS: 23.4° ± 10.0° vs. 21.1° ± 13.9° (*p* = 0.73); mean PI: 49.2° ± 12.7° vs. 54.2° ± 11.8° (*p* = 0.40).

Postoperatively, the mean SVA was 7.6 ± 50.3 mm in the N group and 30.6 ± 31.6 mm in the L group (*p* = 0.21), while the mean TK was 31.8° ± 10.7° in the N group compared with 36.2° ± 12.4° in the L group (*p* = 0.47); mean LL was 46.9° ± 17.3° in the N group and 47.3° ± 8.4° in the L group (*p* = 0.94); mean PT was 14.1° ± 12.0° in the N group and 16.6° ± 9.8° in the L group (*p* = 0.63); mean SS was 32.2° ± 15.5° in the N group and 31.9° ± 8.1° in the L group (*p* = 0.95); mean PI was 46.3° ± 12.7° in the N group and 48.7° ± 8.9° in the L group (*p* = 0.83). At five years postoperative, the mean SVA was 31.0 ± 61.2 mm in the N group and 48.3 ± 44.6 mm in the L group (*p* = 0.48); mean TK was 37.5° ± 5.3° in the N group compared with 41.6° ± 14.8° in the L group (*p* = 0.55); mean LL was 47.9° ± 14.8 ° in the N group and 44.4° ± 7.7° in the L group (*p* = 0.46); mean PT was 18.2° ± 7.0° in the N group and 22.2° ± 9.9° in the L group (*p* = 0.40); mean SS was 32.9° ± 8.9° in the N group and 30.0° ± 8.5° in the L group (*p* = 0.51); and the mean PI was 51.1° ± 14.0° in the N group and 52.3° ± 9.9° in the L group (*p* = 0.83). Both groups achieved a marked reduction in SVA and improvements in TK and LL immediately after the surgery, and these alignments remained stable at the 5-year mark. Despite a tendency for spinal parameters to regress slightly over time, the changes were not statistically significant (Table 4). SAIs might be useful to keep the spinal correction rate regardless of their loosening.

## 5. Discussion

In this study, screw loosening was observed in 78% of patients over a 5-year follow-up period; however, screw loosening did not significantly affect long-term clinical (measured by JOABPEQ) or radiographic (sagittal alignment parameters) outcomes. Typically, an SAI does not fuse the sacroiliac joint, so the screw might become loosened over time; that is, the sacroiliac joint motion remains functional in all directions even after the screw fixation [14,15]. Screw-based sacroiliac joint fixation showed relatively good results, and the technique might reduce the SAI loosening [16]. Despite the frequency of screw loosening in ASD surgery, SAI screws have gained popularity in ASD surgery owing to their ability to provide strong spinopelvic fixation while minimizing complications related to hardware prominence, a common issue with traditional iliac screws [17,18]. Previous studies by Nakashima et al. [5] and Iijima et al. [6] highlighted the biomechanical advantages of SAI screws, including their alignment with the S1 screw and reduced need for extensive hardware. However, the high rates of loosening observed in this study suggest that while SAI screws offer substantial short-term benefits, their long-term stability, particularly in older patients, remains a concern. Interestingly, the lack of significant differences in postoperative outcomes between the two study groups (with and without screw loosening) raises important questions about the clinical relevance of radiographic findings. The absence of a negative impact on clinical outcomes, despite the high incidence of loosening, indicates the limited clinical significance of this radiographic finding, especially when the stability and sagittal alignment of the overall construct are preserved. Furthermore, this might be due to the compensatory load distribution across the adjacent hardware components. A recent study by Zhao et al. [10] and Baron et al. [15] also reported that SAI screw loosening may not correlate with poor clinical outcomes if the surgical construct remains stable. Regarding buttock pain with use of the iliac screw, Brian et al. [19] and Tsuchiya et al. [20] reported a mean numeric rating scale score of 6.9 ± 1.8 at the time of reoperation and 3.1 in the group with iliac screw loosening. In the current study, the buttock pain VAS at 5 years postoperative (N vs. L: 24.6 ± 24.8 vs. 32.6 ± 27.5) was comparable to the previous study, and there was no reoperation.

Presumably, the high incidence of screw loosening observed in this study could be related to patient-specific factors, such as age and bone quality. Older patients, who are more susceptible to osteoporosis and decreased bone density, may have a higher risk of screw loosening due to reduced bone purchase [21]. The biomechanical properties of SAI screws, while advantageous in many cases [22,23], may not completely mitigate the risk of loosening in patients with compromised bone quality. Because the importance of perioperative medical treatment of spine surgery with osteoporosis has been emphasized, strict therapeutic intervention for osteoporosis might be important [24]. Nevertheless, the overall correction achieved during surgery was maintained over the follow-up period, which was highlighted in the lack of significant differences in the clinical outcomes between the groups.

Furthermore, it is possible that other factors, such as patient activity levels or comorbidities, may have contributed to the clinical outcomes observed, irrespective of screw loosening. Future studies should explore these factors to adequately understand the long-term implications of SAI screw loosening and investigate alternative fixation strategies like bilateral multiple SAI screws [25] and adjunctive measures to enhance the long-term stability of SAI screws in patients with compromised bone quality.

This study has several limitations. First, the small sample size and retrospective design may limit the generalizability of our results and introduce potential selection bias. Lack of statistical power may have prevented legitimate comparison of clinical scores between the two groups. In the future, a large number of patients should be included in this study. Second, the 5-year follow-up period may not adequately capture longer-term outcomes. For long-term evaluation of complications and progression of instability, a longer observation is needed in a future study. Third, this study lacks information on the patients’ activity level after surgery, so future studies should include an assessment of this point with additional questionnaires or gait analysis. Fourth, the severity of screw loosening is not considered. It might affect clinical outcomes. Fifth, a comparison cohort with other screws is absent, so the comparison of those cohorts is needed in future studies. Despite these limitations, the study provides important insights; further research with larger samples from multiple centers and longer follow-up periods is needed to validate these findings.

## 6. Conclusions

SAI screw loosening is a frequent but clinically benign occurrence in ASD corrective surgery. The presence of screw loosening does not appear to detrimentally affect long-term clinical outcomes or the maintenance of sagittal alignment, suggesting that the choice of fixation should be individualized based on patient factors such as age and bone quality.

## Figures and Tables

**Table 1:** Patient characteristics and operative data.

Variable	N Group (No Loosening, n = 6)	L Group (Loosening, n = 21)	*p*-Value
Age (years)	69.2 ± 2.3	72.3 ± 7.5	0.03
Sex (female)	2	18	0.06
BMI	23.9 ± 3.4	23.0 ± 3.7	0.63
Femoral T-score	0.60 ± 1.8	−1.08 ± 1.4	0.06
Operative time (min)	631.2 ± 105.2	625.9 ± 136.7	0.94
Intraoperative blood loss (mL)	1795.2 ± 976.3	1769.2 ± 842.2	0.95
L5/S bony fusion (5 years)	5 (83.3%)	17 (81.0%)	0.56
Implant failure	2 (33.3%)	3 (14.3%)	0.24

**Table 2:** Preoperative and postoperative JOABPEQ scores.

Variable	N Group (No Loosening, n = 6)	L Group (Loosening, n = 21)	*p*-Values
Preoperative JOABPEQ Scores			
Pain-related disability	25.0 ± 13.9	41.7 ± 31.4	0.32
Lumbar function	33.3 ± 35.4	54.8 ± 22.5	0.16
Walking ability	30.3 ± 25.7	14.3 ± 27.2	0.31
Social life function	20.3 ± 22.4	25.4 ± 23.2	0.70
Psychological well-being	32.8 ± 13.9	36.6 ± 25.3	0.78
VAS (low back pain)	65.0 ± 41.9	68.8 ± 30.0	0.82
VAS (pain in buttocks and lower limbs)	76.6 ± 23.0	69.3 ± 29.5	0.61
VAS (numbness in buttocks and lower limbs)	76.6 ± 26.3	66.1 ± 34.2	0.53
**Postoperative JOABPEQ Scores (5 years)**			
Pain-related disability	39.3 ± 44.3	44.9 ± 34.0	0.79
Lumbar function	35.5 ± 22.0	32.7 ± 24.9	0.84
Walking ability	41.0 ± 14.9	31.9 ± 34.2	0.65
Social life function	49.3 ± 11.8	38.7 ± 20.6	0.35
Psychological well-being	46.3 ± 9.2	42.7 ± 18.3	0.72
VAS (low back pain)	23.4 ± 16.5	28.4 ± 26.4	0.70
VAS (pain in buttocks and lower limbs)	24.6 ± 24.8	32.6 ± 27.5	0.56
VAS (numbness in buttocks and lower limbs)	56.8 ± 22.8	27.2 ± 21.0	0.01

**Table 3:** Comparison of 5-year postoperative JOABPEQ, SAI loosening, and spinal parameters with L5/S bony fusion.

Variable	Fusion Group (n = 22)	Non-Fusion Group (n = 5)	*p*-Values
Postoperative JOABPEQ Scores (5 years)			
Pain-related disability	38.0 ± 54.1	44.8 ± 32.8	0.77
Lumbar function	52.7 ± 29.0	29.5 ± 21.5	0.12
Walking ability	45.3 ± 48.6	31.6 ± 27.4	0.50
Social life function	50.7 ± 15.5	39.1 ± 19.7	0.36
Psychological well-being	56.0 ± 10.5	41.0 ± 16.6	0.16
VAS (low back pain)	29.1 ± 22.9	18.0 ± 34.7	0.42
VAS (pain in buttocks and lower limbs)	32.9 ± 25.8	21.0 ± 33.3	0.43
VAS (numbness in buttocks and lower limbs)	37.2 ± 23.4	11.8 ± 16.5	0.05
SAI loosening	17 (77.3%)	4 (80.0%)	0.56
Sagittal vertical axis (mm)	44.1 ± 50.3	48.5 ± 33.5	0.87
Thoracic kyphosis (degrees)	41.4 ± 12.4	37.7 ± 20.4	0.62
Lumbar lordosis (degrees)	45.4 ± 10.1	43.5 ± 3.5	0.72
Pelvic tilt (degrees)	19.1 ± 7.7	33.4 ± 8.8	0.003
Sacral slope (degrees)	30.6 ± 8.9	30.8 ± 7.3	0.97
Pelvic incidence (degrees)	49.7 ± 8.7	64.1 ± 12.8	0.01

**Table 4:** Preoperative and postoperative sagittal alignment.

Variable	N Group (No Loosening, n = 6)	L Group (Loosening, n = 21)	*p*-Values
**Preoperative Sagittal Alignment Parameters**			
Sagittal vertical axis (mm)	67.8 ± 43.3	98.4 ± 61.5	0.30
Thoracic kyphosis (degrees)	25.2 ± 17.6	23.6 ± 12.3	0.82
Lumbar lordosis (degrees)	25.9 ± 17.7	22.0 ± 16.1	0.64
Pelvic tilt (degrees)	26.2 ± 6.5	32.3 ± 8.4	0.14
Sacral slope (degrees)	23.4 ± 10.0	21.1 ± 13.9	0.73
Pelvic incidence (degrees)	49.2 ± 12.7	54.2 ± 11.8	0.40
**Postoperative Sagittal Alignment Parameters**			
Sagittal vertical axis (mm)	7.6 ± 50.3	30.6 ± 31.6	0.21
Thoracic kyphosis (degrees)	31.8 ± 10.7	36.2 ± 12.4	0.47
Lumbar lordosis (degrees)	46.9 ± 17.3	47.3 ± 8.4	0.94
Pelvic tilt (degrees)	14.1 ± 12.0	16.6 ± 9.8	0.63
Sacral slope (degrees)	32.2 ± 15.5	31.9 ± 8.1	0.95
Pelvic incidence (degrees)	46.3 ± 12.7	48.7 ± 8.9	0.83
**Postoperative Sagittal Alignment Parameters (5 years)**			
Sagittal vertical axis (mm)	31.0 ± 61.2	48.3 ± 44.6	0.48
Thoracic kyphosis (degrees)	37.5 ± 5.3	41.6 ± 14.8	0.55
Lumbar lordosis (degrees)	47.9 ± 14.8	44.4 ± 7.7	0.46
Pelvic tilt (degrees)	18.2 ± 7.0	22.2 ± 9.9	0.40
Sacral slope (degrees)	32.9 ± 8.9	30.0 ± 8.5	0.51
Pelvic incidence (degrees)	51.1 ± 14.0	52.3 ± 9.9	0.83

## Data Availability

Data are contained within the article.
